# Acute Bacterial Meningitis in Healthy Adult Patients: A Prospective Cohort Study

**DOI:** 10.3390/jcm12113624

**Published:** 2023-05-23

**Authors:** Virginia Pomar, Natividad Benito, Pol Duch, Marc Colomé, Alba Rivera, Pere Domingo

**Affiliations:** 1 Infectious Diseases Unit, Department of Internal Medicine, Hospital de la Santa Creu i Sant Pau—Institut d’Investigació Biomèdica Sant Pau, Universitat Autònoma de Barcelona, 08025 Barcelona, Spain; 2 The University of Queensland, UQ Centre for Clinical Research, Herston, QLD 4029, Australia; 3 CIBERINFEC, ISCIII, 28029 Madrid, Spain; 4 Internal Medicine Department, Hospital de la Santa Creu i Sant Pau, 08025 Barcelona, Spain; 5 Department of Clinical Microbiology, Hospital de la Santa Creu i Sant Pau—Institut d’Investigació Biomèdica Sant Pau, Universitat Autònoma de Barcelona, 08025 Barcelona, Spain

**Keywords:** spontaneous meningitis, bacterial meningitis, cerebrospinal fluid analysis, healthy patient

## Abstract

Spontaneous bacterial meningitis (BM) is more common among patients with underlying conditions, but its characteristics in previously healthy patients are not well described. We analyzed the time trends of BM in terms of characteristics, and outcomes in patients without comorbidities. Patients and methods: Single-center, prospective observational cohort study of 328 adults with BM hospitalized in a tertiary university hospital in Barcelona (Spain). We compared the features of infections diagnosed in 1982–2000 and 2001–2019. The main outcome measure was in-hospital mortality. Results: The median age of the patients increased from 37 to 45 years. Meningococcal meningitis significantly diminished (56% versus 31%, *p* < 0.000) whereas listerial meningitis increased (1.2% versus 8%, *p* = 0.004). Systemic complications were more common in the second period, although mortality did not vary significantly between periods (10.4% versus 9.2%). However, after adjusting for relevant variables, infection in the second period was associated with lower risk death. Conclusions: Adult patients without underlying comorbidities that developed BM in recent years were older and more likely to have pneumococcal or listerial infections and systemic complications. In-hospital death was less likely in the second period, after adjusting for risk factors of mortality.

## 1. Introduction

Bacterial meningitis (BM) has been classically viewed as a disease of extreme ages of life. In these extreme age groups, BM is often associated with the presence of comorbid conditions which may act as predisposing conditions or may synergistically act with CNS infection to worsen the disease prognosis [1,2,3]. In most large series of BM in adult patients, those with predisposing conditions or comorbidities usually represent between 50% and 90% of patients, depending on the BM aetiologic agent [4,5,6,7,8,9,10,11,12,13,14]. Notwithstanding, BM may also affect previously healthy adults, and this part of the disease spectrum often forecasts the most dramatic landscape [1,15,16]. *Neisseria meningitidis*, *Haemophilus influenzae*, and *Streptococcus pneumoniae* account for the overwhelming majority of cases in this population segment and contribute decisively to the still high toll of morbidity and mortality associated with the disease. The development and deployment of effective vaccines against classical meningeal pathogens have led to dramatic decreases in the burden of the disease and changes in BM etiology [15,17,18].

The aim of this study is to assess the spectrum and changes observed in a prospective cohort of adult patients without comorbidity diagnosed with BM between 1982 and 2019.

## 2. Materials and Methods

### 2.1. Setting and Study Population

This is a prospective, observational cohort of all patients with primary BM (not related with prior trauma or surgery) at the Hospital de la Santa Creu i Sant Pau, a large university hospital in Barcelona, Spain. All cases of BM since 1982 are prospectively recorded and followed. Cases of viral, fungal, or mycobacterium meningitis are excluded. Methods of the cohort have been described in detail previously [9,13,17,18]. For the present study, patients over 16 years without underlying conditions and with BM diagnosed between January 1982 and December 2019 were included.

BM was defined as the sudden onset of fever, headache, stiff neck, altered mental status, or focal neurological symptoms, as well as cerebrospinal fluid (CSF) white blood cell (WBC) count >100 cells/µL, hypoglycorrhachia, and/or hyperproteinorrachia, whether or not with the isolation of a pathogenic bacterium from CSF culture or detection of bacterial DNA from CSF. Other definitions are described in previous articles [9,13,17,18].

Sociodemographic data, symptoms and signs on admission, laboratory results, radiological examination, treatment, and outcome were prospectively collected with a standard case report form. Data on mortality were obtained by monitoring patients during hospitalization.

The cohort was divided into two periods: 1982–2000 (period 1) and 2001 to 2019 (period 2).

The study was approved by the hospital’s ethics committee. Written informed consent was obtained from all participating patients or their legal representatives.

### 2.2. Microbiology Methods

CFS specimens were processed according to standard microbiological procedures, including Gram stain and culture [19]. Multiplex PCR using the BioFire FilmArray^®^ Meningitis/Encephalitis (ME) PCR Panel (Biomerieux, Salt Lake City, USA) was implemented in the routine in September 2016 and from that date was performed according to the manufacturer’s protocol and the laboratory diagnostic algorithm on CSF samples with ≥100 cells/mm^3^. The panel was used to detect 14 pathogens, including the following bacterial species: *Escherichia coli*, *H. influenzae*, *Listeria monocytogenes*, *N. meningitidis*, *Streptococcus agalactiae,* and *S. pneumoniae*. Blood cultures were performed by conventional systems, BacT/Alert 3D or Virtuo (BioMérieux, Durham, CA, USA). Isolates obtained from routine cultures were identified using standard methods and MALDI-TOF (Bruker, Bremen, Germany).

### 2.3. Statistical Analysis

Continuous variables were described with medians and interquartile range (IQR) and qualitative variables as percentage of the total included cases. Categorical data were compared using the Chi-square test (or Fisher’s exact test when appropriate) and continuous data with the *t*-test or Mann–Whitney test. A multivariate logistic regression analysis was performed to identify factors independently associated with death during hospitalization. Covariates were chosen based on previous research and clinical judgment, but also, with the univariate analysis results. All tests were 2-tailed, and *p* < 0.05 was considered significant. Statistical analyses were performed using SPSS statistical software, version 22 (IBM Corp., Armonk, NY, USA).

## 3. Results

From 1982 to 2019, we prospectively included 837 episodes of spontaneous or primary BM. Three hundred and twenty-eight episodes of BM were diagnosed in 320 previously healthy patients (38%). Eight patients had a recurrence of BM. The number of BM cases decreased between the two analyzed periods, 1982–2000 and 2001–2019, 241 (73%) and 87 (27%), respectively (*p* < 0.001).

Two hundred (61%) were women. Overall median age was 35 years (IQR, 19–56), although patients were older in the most recent years (Table 1). Patients in period 2 presented less frequently neck stiffness, headache, rash, and the meningeal triad (neck stiffness, fever, and altered mental status). The duration of symptoms before admission to emergency room was shorter (42 vs. 38 h, *p* < 0.377), although the difference was not statistically significant. However, median time until appropriate antibiotic initiation was significantly longer (3 vs. 11 h, *p* < 0.003) in the second period. A distant focus was identified in 104 of the 328 episodes (32%), but more commonly in the recent time interval. Upper respiratory tract infection (70 episodes, 21%), otitis or sinusitis (35 episodes, 11%), and pneumonia (14 episodes, 4%) were the most frequent extrameningeal foci of infection.

In our cohort, only eight patients (3.3%) without comorbidities underwent brain computed tomography (CT) in the emergency department in period 1, compared with 62 patients (71.3%) in the second period.

The clinical and laboratory characteristics of study patients are shown in Table 1 and Table 2.

Lumbar puncture was performed in all patients. CSF culture was positive in 234 (71%) and blood culture in 122 (37%) cases (Table 2). The most common isolated organism was *N. meningitidis* 163 (50%), followed by *S. pneumoniae* 66 (20%). Although cyto-biochemical findings were similar between the two periods, meningococcal meningitis significantly decreased whereas meningitis caused by *L. monocytogenes* significantly increased.

Laboratory characteristics were similar between the 1982–2000 and 2001–2019 patients.

In the hospital, most patients initially received monotherapy with penicillin (149 [45%], followed by a third-generation cephalosporin (122 [37%]). In the second period, the first choice was third-generation cephalosporin, adopted by 48 patients (54%). Median duration of antibiotic treatment was 12 days (IQR 8–14), longer in the second period: 10 days (IQR, 8–14) vs. 14 days (IQR, 10–14), *p* < 0.000. One hundred and three patients (31%) received adjunctive steroids before or together with the first dose of antibiotic treatment, most of them in the second period (Table 3).

Overall mortality rate was 10% (33 patients), and 30 surviving patients had a neurological deficit at discharge. There were no differences in mortality between periods, but in the second period patients had more often acute respiratory failure, acute kidney failure and septic shock, and more patients needed vasoactive drugs, dialysis, and mechanical ventilation. Independent factors associated with higher mortality in multivariate analysis included septic shock (OR = 14.520, *p* < 0.001), acute renal failure (OR = 6.933, *p* < 0.007), and age 65 or older (OR = 3.766, *p* < 0.029) (Table 4). The use of appropriate empirical treatment and having the infection in the second period were associated with less mortality.

## 4. Discussion

Spontaneous bacterial meningitis is more frequent in patients with comorbidities, such as cancer, diabetes, liver disease, kidney disease [6,7,8,9,10,11,12,13,14,20]. However, our study highlights that, although much more infrequently and with a decreasing prevalence over time, acute BM still hits previously healthy people. In our study they represent a substantial population segment in our cohort (40%), a proportion which decreased between the periods into which the study was divided (49.6% vs. 24.8%, *p* < 0.001).

Acute BM in this population is especially dramatic with death and secondary disabilities, given its young age, absence of prior disabilities, and longer life expectancy. All these circumstances result in a high loss of quality-adjusted life years and are sometimes accompanied by social alarm and distress, especially in cases of epidemic meningitis [1,3,21].

The epidemiology of BM has substantially changed during the last quarter of the 20th century thanks to the progressive development and deployment of highly effective vaccines against bacterial meningeal pathogens [18,20]. However, *N. meningitidis* and *S. pneumoniae* still ranked first among the causes of bacterial meningitis in the first two decades of the 21st century, even though their vaccines have been available for decades [22]. This is probably because the most frequent meningococcal serogroup causing invasive disease in Spain has always been serogroup B and its effective vaccine was not available until 2013, and it has still not been included in children’s vaccination schedule in our country [23]. Likewise, pneumococcal vaccination has been for many years performed with non-conjugate pneumococcal vaccines [21] and it has never been widely used in Spain, except for immunosuppressed patients, those with severe comorbidities and, more recently, the elderly [24]. The appearance of highly effective conjugate pneumococcal vaccines will change the landscape forever, although they were not available until 2000 and they were included in the vaccination schedule in 2003 [23].

Although we did not collect the vaccination of each patient, the slow escalation of vaccine deployment may partly explain the steady decrease in the number of cases of potentially avoidable bacterial meningitis [18,20,22,23,25,26]. Consequently, during the second period of the study, meningeal pathogens, such as *L*. *monocytogenes*, possibly due to ready-to-eat foods and the higher age of subjects, increased significantly, without replenishing yet the ecological niche of meningococci and pneumococci.

Most patients in our cohort did not present with the classical meningeal triad significantly more frequently during the second study period, which is striking since there were no immunosuppressed or comorbid patients, two well-known causes of blurred clinical symptomatology. It also does not seem to relate to a different interval from onset of symptoms to hospital admission, or to the administration of antibiotic therapy before admission, since both parameters were comparable between periods. However, the number of patients older than 65 years was higher in the second period, and atypical presentation is a hallmark of ABM in the elderly [17].

The lack of clinically suggestive symptomatology may have contributed to the longer interval between admission and appropriate antibiotic therapy, which almost doubled in the second period. Moreover, cranial CT was most frequently performed during the second period, and this delayed the spinal tap and consequently the start of antibiotic therapy which carries a worse prognosis [27,28,29,30,31,32,33]. Current international guidelines have proposed the “red flags” for identifying patients that need a cranial [34]. Following these guidelines, most of the cerebral computed tomography performed in our patients was unnecessary.

The number and type of neurologic complications were similar during both periods. But systemic complications were more frequent in patients in the second period. The delay in starting proper antibiotic therapy and the number of patients older than 65 may partly explain this difference in complications. Care for critically ill patients, such as those with acute BM, has improved in recent years, which should have a positive impact on patients’ outcomes during the second period. The widespread use of dexamethasone in the second period could also provide a benefit. However, the beneficial effects of dexamethasone are restricted to pneumococcal meningitis in adults and to neurologic sequelae in *H. influenzae* meningitis in children [2,3,34]. Moreover, in our series, 75% of episodes included were of non-pneumococcal etiology.

Systemic complications of acute BM are independent predictors of a bad outcome in our study, together with age, whereas having meningitis in period 2 and an adequate empiric antibiotic therapy were protective factors. This analysis highlights the prognostic importance of age, even in the absence of comorbidities or immunosuppression, suggesting that immunosenescence plays a role in predisposition to an evolving trend of BM [17], a point shared with many other infections caused by capsulated bacteria such as meningeal pathogens. Having acute BM during the second period represents a survival advantage, although the case-fatality rate was similar in crude analysis. As noted, patients in the second study period were older and had a higher prevalence of systemic complications, both of which are independent predictors associated with higher mortality rates. The meningeal pathogens per se, despite the virulence differences between them, do not seem to play a role concerning mortality.

Our study had several limitations, principally the fact that it was a single center study. Second, over these almost four decades, changes have taken place in identifying the bacterial causes and in the management of critical neurological patients, whose effect on mortality can be difficult to evaluate.

## 5. Conclusions

In summary, previously healthy people can have BM, predominantly caused by *N. meningitidis* and *S. pneumoniae*, which often presents with shadowed clinical manifestations, but *L. monocytogenes* is also found in healthy patients.

## Figures and Tables

**Table 1:** Clinical and laboratory characteristics on admission of adults with bacterial meningitis.

Characteristics	Cohort 1982–2000 (*n* = 241)	Cohort 2001–2019 (*n* = 87)	*p* Value
Male sex	90 (37.3)	38 (43.7)	0.181
Age–years, median (IQR)	37 (36)	45 (39)	<0.001
• ≤18 years–*n* (%)	62 (25.7)	10 (11.5)	0.008
• 18–49 years–*n* (%)	103 (42.7)	37 (42.5)	
• ≥50 years–*n* (%)	76 (31.5)	40 (46)	
• ≥60 years–*n* (%)	35 (14.5)	23 (26.4)	
Distant focus infection	67 (27.8)	37 (42.5)	0.009
Route of acquisition			
• Hospital-acquired (vs. community-acquired)	4 (1.7)	1 (1.1)	1.000
Symptoms on presentation			
• Fever	232 (96.3)	83 (95.4)	0.640
• Altered mental status	148 (61.4)	44 (50.6)	0.147
• Neck stiffness	208 (86.3)	69 (79.3)	0.025
• Triad of fever, neck stiffness, and change in mental status	131 (54.4)	30 (34.5)	0.005
• Headache	225 (93.4)	61 (70.1)	<0.001
• Nausea and/or vomiting	180 (74.7)	56 (64.4)	0.128
• Focal neurological deficits	35(14.5)	9 (10.3)	0.365
• Coma	27(11.2)	11 (12.6)	0.700
• Seizures	6 (2.5)	9 (10.3)	0.293
• Rash	108 (44.8)	25 (28.7)	0.020
• Systolic blood pressure–mm Hg (SD)	120 (30)	122 (27)	0.587
• Diastolic blood pressure–mm Hg (SD)	71 (20)	73 (16)	0.592
Interval symptoms-admission, hours (IQR)	42 (24)	38 (39)	0.377
Interval admission-therapy, hours (IQR)	3 (2)	11 (7)	0.003
Prior antimicrobial therapy	74 (30.7)	21 (24.1)	0.416
Cerebral computed tomography	8 (3.3)	62 (71.3)	<0.001
White blood cell count, median (IQR)	17,074 (11,350)	18,190 (11,400)	0.274
Platelet count/mm^3^, median (IQR)	197,890 (110,000)	490,419 (136,250)	0.122

IQR = interquartile range; SD = standard deviation. Values are reported as no./no. evaluated (%), unless otherwise noted.

**Table 2:** CSF findings, microbiologic features and aetiology of bacterial meningitis in adult patients.

Characteristics	Cohort 1982–2000 (*n* = 241)	Cohort 2001–2019 (*n* = 87)	*p* Value
CSF examination			
• White blood cell count/mm^3^, median (IQR)	2346 (2444)	2705 (3956)	0.395
• Protein, g/L, median (IQR)	4.3 (4.7)	4.1 (4.7)	0.611
• CSF/plasma glucose ratio, median (IQR)	0.26 (0.39)	0.28 (0.38)	0.545
Positive CSF Gram-stained smear	123 (51)	28 (32.2)	0.03
Positive CSF culture	176 (73)	58 (66.7)	0.164
Positive blood culture	85 (35.3)	37 (42.5)	0.118
Aetiology			
• Meningococcal	136 (56.4)	27 (31.0)	<0.001
• Pneumococcal	45 (18.7)	21 (24.1)	0.279
• *Listeria monocytogenes*	3 (1.2)	7 (8.0)	0.004
• Gram-negative bacilli ^1^	9 (3.7)	3 (3.4)	1.000
• *Haemophilus influenza*	7 (2.0)	1 (1.1)	0.686
• Other ^2^	4 (1.7)	9 (9.2)	0.004
• Unknown origin	36 (14.9)	20 (23.0)	0.097

Gram-negative bacilli ^1^: *Escherichia coli* (9), *Citrobacter freundii* (1), *Pseudomonas* spp. (1), *Serratia marcescens* (1). Other ^2^: *Streptococcus agalactiae* (2), *Staphylococcus aureus* (4), *Streptococcus bovis* (1), *Streptococcus pyogenes* (2), *Streptococcus viridans* (2), *Brucella* spp. (1), Group G Streptococcus (1). IQR = interquartile range; CSF: cerebrospinal fluid. Values are reported as no./no. evaluated (%), unless otherwise noted.

**Table 3:** Evolving features and outcome of bacterial meningitis.

Characteristics	Cohort 1982–2000 (*n* = 241)	Cohort 2001–2019 (*n* = 87)	*p* Value
Neurological complications	37 (15.4)	16 (18.4)	0.208
• Coma	27 (11.2)	11 (12.6)	0.700
• Seizures	6 (2.5)	9 (10.3)	0.015
• Focal neurological deficits	35 (14.5)	9 (10.3)	0.365
• Cranial palsies	7 (6.6)	28 (4.6)	0.337
Systemic complications	47 (19.5)	25 (28.7)	0.096
• Acute respiratory failure	17 (7.1)	26 (29.9)	<0.001
• Acute kidney failure	13 (5.4)	13 (14.9)	0.009
• Septic shock	29 (12.0)	19 (21.8)	0.033
• Disseminated intravascular coagulation	0	0	
• Rhabdomyolysis	10 (4.1)	4 (4.6)	1.000
Therapeutics			
• Adequate empiric antibiotic therapy	227 (94.2)	85 (95.4)	0.789
• Dexamethasone therapy	52 (21.6)	51 (58.6)	<0.001
• Vasoactive drugs	25 (10.4)	18 (20.7)	0.025
• Mechanical ventilation	20 (8.3)	23 (26.4)	<0.001
• Dialysis	1 (0.4)	6 (6.9)	0.002
Outcome			
• Neurological sequelae	7 (2.9)	3 (3.4)	0.728
• In-hospital mortality	25 (10.4)	8 (9.2)	0.838
Meningococcal	6 (4.4)	1 (3.7)	1.000
Pneumococcal	9 (20)	4 (19)	1.000
Listerial	1 (33)	0	0.300
Gram-negative bacilli	5 (55.6)	0	0.205

Values are reported as no./no. evaluated (%), unless otherwise noted.

**Table 4:** Multivariate analysis for effect on unfavorable outcome.

Variable	Odds Ratio	95% CI	*p* Value
**Septic shock**	**14.520**	**3.504–60.167**	**<0.001**
**Acute renal failure**	**6.933**	**1.693–28.382**	**0.007**
**Age (≥65 years)**	**3.766**	**1.142–12.415**	**0.029**
Positive CSF culture	2.111	0.286–15.579	0.464
Positive blood culture	1.320	0.412–4.227	0.640
Triad of fever, neck stiffness, and change in mental status	1.222	0.396–3.769	0.728
White cell count in CSF > 1000/mm^3^	0.700	0.233–2.110	0.527
Period 2	0.169	0.041–0.708	0.015
Adequate empiric antibiotic therapy	0.047	0.008–0.279	0.001
Aetiology			
• Neisseria meningitidis	1.000 (reference)		
• Streptococcus pneumoniae	2.403	0.354–16.332	0.370
• Listeria monocytogenes	0.835	0.033–21.199	0.913
• Gram-negative bacilli	1.137	0.127–10.180	0.908

CI = confidence interval. Nagelkerke’s R^2^ for the adjusted model = 0.446. Variables that are statistically significant are shown in bold type.

## Data Availability

The data will be made available to researchers on an individual basis upon request.
